# Enhancing the Mechanical Properties of Carbon Fiber/Epoxy Composites by Constructing a “Three-Dimensional Nanospider Web” Rigid–Flexible Interface Layer

**DOI:** 10.3390/ma19173685

**Published:** 2026-08-30

**Authors:** Xiaoda Wei, Yi Bian, Kang Jin, Ruiling Lv, Wenkang Yi, Ruina Ma, Xue Zhao, Mingxu Yang

**Affiliations:** 1 State Key Laboratory of High Performance Roll Materials and Composite Forming, Hebei Key Laboratory of New Functional Materials, School of Materials Science and Engineering, Hebei University of Technology, Tianjin 300401, China; wei17349869951@163.com (X.W.);; 2 State Key Laboratory of Advanced Molding Technology and Equipment, China Academy of Machinery Science and Technology Group Co., Ltd., Beijing 100048, China; 3 China Machinery Industry Technology Research Institute of Precision Forming, Wuhu 241000, China; 4 School of Mechanical Engineering, Hebei University of Technology, Tianjin 300401, China

**Keywords:** carbon fiber, surface modification, mechanical properties, modulus transition

## Abstract

To enhance the mechanical properties of carbon-fiber-reinforced polymer composites (CFRPs), this study devised a novel three-dimensional web-like “rigid–flexible” surface modification strategy. The synergistic incorporation of carbon nanotubes (CNTs), polydopamine (PDA), and cellulose nanofibers (CNFs) constructed a “three-dimensional nanospider web” modulus transition layer. The modified carbon-fiber (CF-0.1%CNT-PDA-CNF) surface exhibits a three-dimensional network structure, with significantly increased surface roughness. The surface energy increased by 128.60% compared to the desized carbon fiber, thereby improving the wettability of the carbon-fiber surface. The results of both PeakForce-Quantitative Nanomechanical Mapping (PF-QNM) and EDS analyses indicate that a transition layer of a certain thickness initially formed at the interface. At the interface, the modulus exhibits a gradual gradient decrease from carbon fiber to epoxy resin, achieving more efficient stress transfer. The interfacial shear strength (IFSS, 95.71 MPa), interlaminar shear strength (ILSS, 73.19 MPa), tensile strength (701.08 MPa), and flexural strength (934.41 MPa) of the CF-0.1%CNT-PDA-CNF/EP composite material increased by 38.39%, 54.93%, 51.74%, and 64.98%, respectively, compared to the composite material made from desized carbon fiber. Through hydrogen bonding, covalent bonding, and π-π interactions, CNTs, CNFs and PDA formed a “rigid–flexible” transition layer with a modulus gradient at the CF-epoxy interface, achieving a significant enhancement in the mechanical properties of the composite material.

## 1. Introduction

Carbon fiber has excellent characteristics, including a high strength-to-weight ratio, low density, and a high modulus, and has found extensive application across multiple sectors, including aerospace, automotive manufacturing, and sports equipment [1,2]. However, the chemical inactivity of carbon-fiber surfaces leads to weak bonding between the carbon fibers and the resin [3,4]. This weak interfacial interaction struggles to efficiently transfer stresses from carbon fiber to the resin [5], readily inducing stress concentration within carbon-fiber composites. Under applied loading conditions, this leads to interfacial debonding and delamination between the carbon fibers and the resin, ultimately causing composite failure [6]. This severely limits their application scope. Therefore, enhancing the interfacial bond strength between carbon fibers and resin is crucial for improving the mechanical properties of carbon-fiber-reinforced composite materials [7].

The surface modification of carbon fibers is a key approach to enhancing their interfacial bonding strength with resin. Currently employed modification techniques include oxidation [8], plasma treatment [9], sizing agent coating [10], in situ self-assembly [11], and chemical grafting [12]. During carbon-fiber modification, the adhesion between carbon fibers and the epoxy resin matrix can be explained by fundamental interfacial theories, such as interfacial chemical bonding theory, mechanical interlocking theory, and modulus transition theory. Upon introducing active functional groups, such as carboxyl groups [13], hydroxyl groups [14], and amino groups [15], onto the carbon-fiber surface, the carbon fibers form chemical bonds with the resin while also enhancing the surface wettability and surface energy. For instance, Zhang et al. [16] first subjected the carbon-fiber surface to oxidation treatment, then grafted amino-functionalized graphene oxide (GO-NH_2_) onto the CF surface via covalent bonding. This modification significantly improved the surface energy of the CFs, ultimately enhancing the IFSS of the composite material by 36.4%. Moreover, non-covalent interactions, such as van der Waals forces, hydrogen bonds, and electrostatic interactions, exist between carbon fibers and the matrix [17], which can further enhance interfacial bonding. Quan et al. [18] built a PDA/CNF/PVA/aminated-CNT synergistic network on carbon fibers mainly through non-covalent physical interactions, where PVA’s abundant hydrogen bonds and covalent bonding jointly enhanced CNF-CNT interactions and improved interfacial performance. In contrast, this work achieved covalent anchoring of CNT backbones onto CF substrates via a high-temperature dehydration–condensation reaction, followed by the use of PDA as a covalent bridge to connect pre-grafted CNTs with CNFs. This hierarchically covalent-bonded architecture generates a continuous “rigid–flexible” modulus gradient interphase. Numerous studies have demonstrated that interfacial CNT incorporation can strengthen mechanical interlocking and suppress relative sliding between fibers and the matrix [19]. Other efforts have attempted to tailor interfacial architecture via functional agents, which build gradient interphases through chemical cross-linking and physical adsorption for more efficient stress transfer [20]. Inspired by the structure of palm trees, Tan et al. [21] constructed a “soft–rigid–soft” architecture on carbon-fiber surfaces using a tannic acid polymer (TA) and polyhedral oligomeric sesquisiloxane (POSS) as flexible layers, with CNTs as the rigid layer. The modified CF-reinforced composite exhibited improvements in IFSS, ILSS, and TFBT of 84.08%, 50.43%, and 80.37%, respectively. These research methodologies effectively enhance interfacial bonding through surface chemical modifications, physical morphology regulation, or reductions in modulus disparity between fibers and resin. Among these methods, the creation of a modulus transition layer between the fibers and the resin represents an effective strategy for enhancing the mechanical properties of composite materials.

By constructing a modulus gradient transition layer between high-modulus carbon fibers and a low-modulus polymer matrix, distinct modulus interface layers can be formed within carbon-fiber composites through the introduction of “flexible” chains, “rigid” chains, and gradient molecules. This approach effectively disperses stresses and optimizes overall mechanical properties [22,23]. Feng et al. [15] reported a dual rigid–flexible interphase using CNTs and polyamide, which enriched surface polar groups, roughness, and wettability. Zheng et al. [24] employed CNTs and polyetheramine (PEA) to fabricate a multi-scale “nunchaku-like” rigid–flexible–rigid architecture on the surface of carbon fibers. The IFSS, ILSS, and tensile bond strength of the composite material were enhanced by 73.4%, 65.4%, and 137.0%, respectively. Pu et al. [25] utilized a gentle self-assembly approach to integrate positively charged polyethyleneimine (PEI) with a negatively charged metal–organic framework of (UIO-66)/graphene oxide (GO) nanocomposites onto carbon-fiber substrates, forming a novel “soft–hard” interfacial layer. Through interfacial nano-scale coupling interactions and multiple crack deflection mechanisms, the IFSS and ILSS of the composite material were enhanced by 42.12% and 23.07%, respectively. The formation of a multiphase interfacial modulus transition layer between carbon fibers and the resin matrix enables a more effective optimization of stress transfer pathways and mitigation of interfacial stress concentration, thereby significantly enhancing the mechanical properties of carbon-fiber composites.

This work develops a modification route that optimizes the modulus gradient, mechanical interlocking, and chemical bonding through an interface design, achieving significant improvements in the mechanical properties of the composites. The interfacial shear strength (IFSS), interlaminar shear strength (ILSS), tensile strength, and flexural strength of modified CF-reinforced composites attained 69.12 MPa, 73.19 MPa, 701.08 MPa, and 934.41 MPa, increasing by 38.39%, 54.93%, 51.74%, and 64.98% relative to unmodified CF/EP composite. This work confirms that the proposed fiber surface modification strategy can effectively enhance composite mechanical properties. It holds promising application potential and provides new perspectives for designing high-performance composites.

## 2. Experiments

### 2.1. Materials

A polyacrylonitrile-based carbon-fiber fabric (T300, average diameter 7 μm, density 1.76 g/cm^3^) was manufactured by Sinosteel Jilin Carbon Co., Ltd., Jilin, China. Carboxylated carbon nanotubes (purity 99%, diameter range 10–15 nm, length approximately 2 μm, density 0.09 g/cm^3^) were procured from Shenzhen Suiheng Technology Co., Ltd., Shenzhen, China. Dopamine hydrochloride (DA, 98% purity) and carboxylated cellulose nanofibers (CNFs, diameter 4–10 nm, length approximately 200 nm) were produced by Shanghai McLean Biochemical Co., Ltd., Shanghai, China. Epoxy resin (E51, molecular weight 350–400, epoxy value 0.51) was manufactured by Guangzhou Hehui Chemical Co., Ltd., Guangzhou, China. The curing agent (T31, amine value 460 ± 20 mg KOH) was manufactured by Shandong Guohua Chemical Co., Ltd., Jinan, China. Tris-HCl (pH = 8.5, analytical grade) was manufactured by Beijing Inokai Technology Co., Ltd., Beijing, China. Acetone, hydrochloric acid, anhydrous ethanol, and diiodomethane were all analytical grade chemicals purchased from Tianjin Chemical Reagents Co., Ltd., Tianjin, China. Deionized water was prepared in-house.

### 2.2. Preparation of a “Three-Dimensional Nanospider Web” Structure on Carbon-Fiber Surfaces

The carbon-fiber fabric was submerged in an acetone solution for a duration of 72 h to eliminate sizing agents and other contaminants. It was then ultrasonically cleaned in deionized water for 15 min. The washed samples were dried in an oven at 80 °C for 6 h, yielding desized carbon fibers (CFs).

We dissolved a specified quantity of carboxylated CNTs in an ethanol solution containing 1.6 g of epoxy resin (E51), with a total solution volume of 800 mL. We subjected the sample to sonication for a duration of 30 min to ensure homogeneous dispersion of the CNTs. Subsequently, CFs were immersed in the aforementioned CNT solution and subjected to ultrasonication for 3 h. The CFs were then removed, rinsed repeatedly with deionized water, dried, and then placed in a tube furnace. Under an atmosphere of high-purity argon at 950 °C, CFs were subjected to the reaction for a duration of 3 h, resulting in carbon fibers modified with CNTs at different concentrations. Under high-purity argon protection, the CF graphite framework remains stable at 950 °C. Combined with SEM, AFM morphology, and surface wettability characterization, no obvious bulk degradation of the CF substrate was observed after thermal treatment.

We weighed 0.97 g of Tris and then dissolved it in 600 mL of deionized water. We added a suitable volume of hydrochloric acid to adjust the pH to 8.5, and we subsequently diluted the solution to a final volume of 800 mL. We dissolved 2.4 g of DA in the prepared Tris-HCl buffer solution. After magnetic stirring for 30 min, we dissolved 0.63 g of CNFs in the solution and continued ultrasonic treatment for 1 h to form a dispersion of DA and CNFs. Subsequently, CFs modified with different concentrations of CNTs were immersed in the dispersion solution for 12 h at room temperature under continuous magnetic stirring, yielding CF-xCNT-PDA-CNF (x = 0%, 0.05%, 0.1%, 0.2%). The values of 0.05%, 0.1%, and 0.2% denote the mass fractions (wt%) of carboxylated CNTs with respect to the total mass of the solution.

### 2.3. Preparation of Carbon Fiber/Epoxy Resin Composites

**Figure 1:**
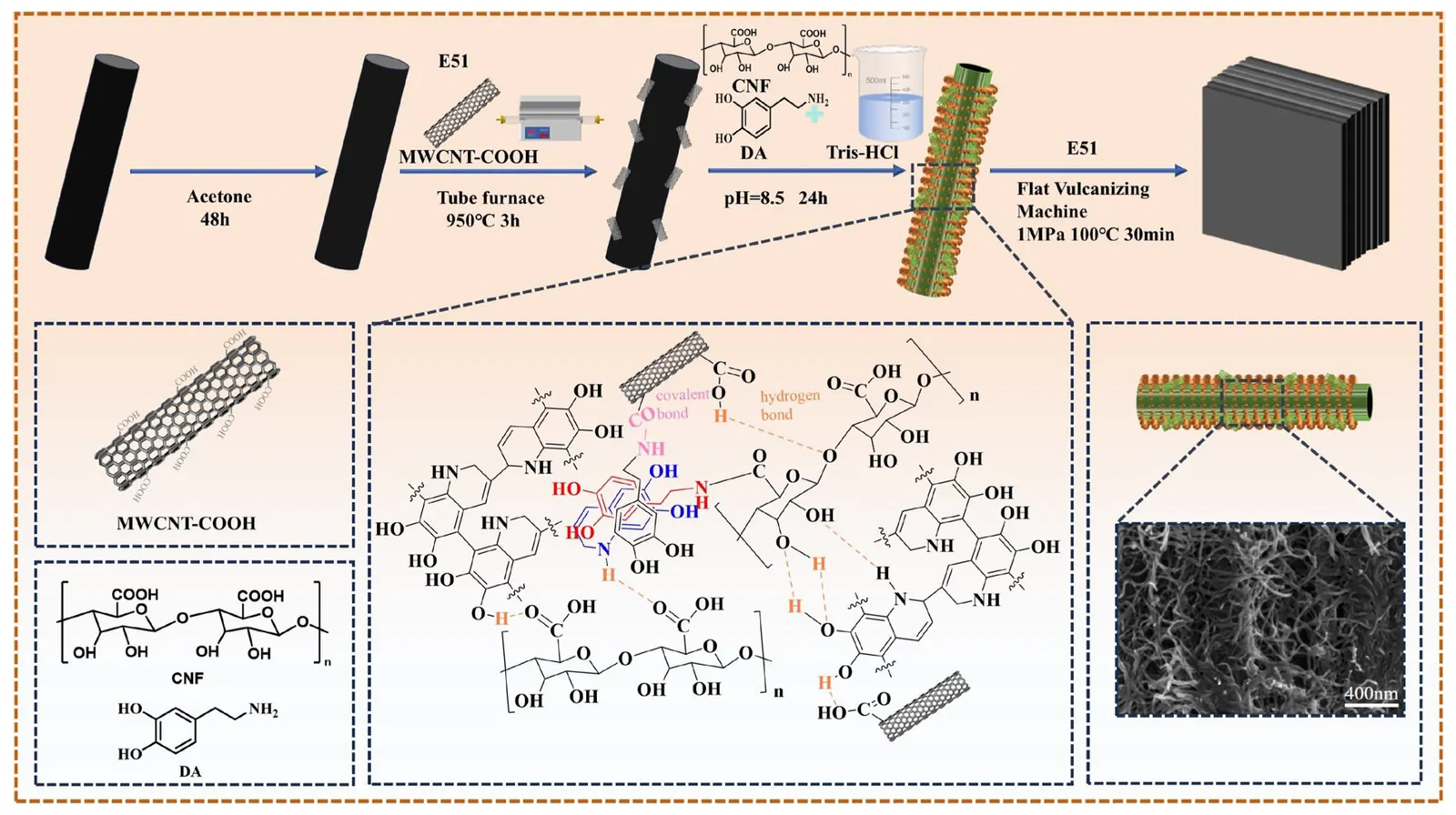
Schematic illustration of constructing a “three-dimensional nanospider web” rigid–flexible structure on a carbon-fiber surface and preparing its composite material.

The epoxy resin underwent ultrasonic degassing treatment and was then mixed with the curing agent in a 4:1 ratio before being placed in a vacuum chamber for further degassing. The carbon fiber volume fraction was maintained at 50 ± 2%. Carbon fibers and resin were layered alternately in the mold, followed by compression molding in a flat plate vulcanizer at 100 °C, 1 MPa, and 30 min to produce the CF/EP composite material. Orthogonal experiments were performed to evaluate the curing efficiency. The results demonstrated that further prolonging the curing time beyond 30 min at 100 °C cannot yield additional mechanical improvement, confirming that the 100 °C/30 min curing schedule is sufficient for full cross-linking of the present epoxy–amine system. Figure 1 illustrates the carbon-fiber surface modification and composite fabrication process. Subsequent machining of the molded sheet yields specimens of the required dimensions for mechanical testing.

Different from prior strategies based on physical blending or one-step deposition of CNT/polymer systems, this work proposes a modification route with three sequential mechanistic stages implemented via two experimental procedures. First, carbon nanotubes (CNTs) are chemically grafted onto carbon-fiber surfaces through a high-temperature dehydration–condensation reaction to form rigid backbones. Second, polydopamine (PDA) acts as a flexible bonding bridge to covalently connect CNT networks with cellulose nanofibers (CNFs). The resulting covalently bonded CNT-PDA-CNF “three-dimensional nanospider web” architecture exhibits a continuous modulus gradient, which, to the best of our knowledge, has not been systematically documented (see Figure 1). This transition layer tailors the modulus gradient at the carbon fiber–resin interface and reinforces both mechanical interlocking and chemical bonding between fibers and the matrix, thus achieving substantial improvements in composite mechanical performance. CNTs assemble into nano-scale whisker-like structures on fiber surfaces, enlarging the specific surface area and offering active sites for the in situ growth of PDA. Carboxyl, hydroxyl, and other functional groups on CNTs further promote chemical interactions with PDA and the epoxy matrix. With a modulus intermediate between CNTs and CNFs, PDA contains abundant reactive catechol and amino moieties. It adheres to fiber surfaces via covalent bonds, hydrogen bonds, and π-π stacking interactions while embedding CNTs and CNFs to realize efficient cross-linking of the three components. Owing to their high flexibility, CNFs form an entangled network with CNTs. The hydroxyl-rich surfaces of CNFs form chemical bonds with PDA functional groups; meanwhile, CNFs possess a favorable chemical compatibility and desirable wettability toward epoxy resin.

### 2.4. Characterization Methods

The surface morphology of carbon fibers and the fracture surface morphology of the composite after failure were characterized by scanning electron microscopy (SEM, Thermo Apreo 2S, Thermo Fisher Scientific, Waltham, MA, USA). SEM observations and EDS elemental line-scan analysis were performed using a Thermo Apreo 2S scanning electron microscope. An accelerating voltage of 5 kV and a working distance of 8 mm were adopted for SEM imaging. The composite samples were sputter-coated with a gold conductive layer at an approximate thickness of 8 nm prior to SEM/EDS measurements. Three independent specimens were examined for each material group, and micrographs were acquired from no fewer than five random observation sites on each specimen, with representative images selected for presentation. Linear scans of the carbon element by energy-dispersive spectroscopy (EDS, Thermo Fisher Scientific, Waltham, MA, USA) were performed to determine the interfacial thickness of the composite. Four independent fiber–matrix interfacial regions were analyzed for each composite, and the reported interfacial thickness value was averaged from these four line-scan profiles. The surface chemical compositions of carbon fibers were analyzed using X-ray photoelectron spectroscopy (XPS, Nexsa G2, Thermo Fisher Scientific, Waltham, MA, USA). Three replicates were tested per modification condition. Spectra were acquired at multiple surface locations, and elemental atomic percentages were averaged from valid data. The Raman spectrum of carbon fiber was obtained by high-resolution micro-Raman spectroscopy (LabRAM HR Evolution, HORIBA Scientific, Longjumeau, France) with a 532 nm excitation source, covering the wavenumber range of 200–2500 cm^−1^. Three sample replicates were measured for each condition, and representative spectra are shown in the manuscript. Prior to calculating the I_D_/I_G_ ratios, all acquired Raman spectra were baseline-corrected using the built-in rubber-band baseline-subtraction algorithm within the supporting software of the LabRAM HR Evolution instrument (HORIBA Scientific, Longjumeau, France). AFM was used to characterize the surface roughness evolution of carbon fibers. Five independent regions were scanned per sample, and the obtained Ra values are expressed as mean ± standard deviation. The composite cross-section was polished to ensure a surface roughness of less than 20 nm. Subsequently, the modulus variation at the composite interface was characterized by atomic force microscopy (AFM, Bruker Dimension Icon) in Peak Force Quantitative Nanomechanical Mapping (PF-QNM) mode. PF-QNM measurements were performed using an RTESPA-300 probe (Bruker, Bruker Corporation, Billerica, MA, USA) with a nominal spring constant of 40 N/m. The peak force was set to 2 nN, and the scan rate was 0.5 Hz. Five distinct cross-section locations were scanned for each composite sample. The reported modulus transition layer width was averaged from multiple extracted line profiles, and representative modulus distribution images were selected. The modulus was extracted using the Derjaguin–Muller–Toporov (DMT) model. The interfacial layer thickness was defined as the distance over which the modulus gradually transitions from 90% of the fiber value to 10% above the matrix value.

The contact angles of carbon fibers with deionized water and diiodomethane were measured using a contact angle measuring instrument (Dataphysics OCA 20, Germany, DataPhysics Instruments GmbH, Filderstadt, Germany) on individual carbon-fiber filaments extracted from the fabric. The droplet volume was kept to a minimum (1 µL), and each sample was measured 10 times to obtain a reliable average value. The surface energy was then calculated based on the following equation [26].

(1) $$\gamma_{l}(1+\cos\theta )=\sqrt{2(\gamma_{l}^{p}\gamma_{f}^{p})}+\sqrt{2(\gamma_{l}^{d}\gamma_{f}^{d})}$$

(2) $$\gamma_{f}=\gamma_{f}^{p}+\gamma_{f}^{d}$$

where $\gamma_{l}$, $\gamma_{l}^{d}$, and $\gamma_{l}^{P}$ represent the surface tension of the immersion liquid and its corresponding dispersion and polar components. Similarly, $\gamma_{f}$, $\gamma_{f}^{d}$, and $\gamma_{f}^{p}$ correspond to the total surface tension, dispersive component, and polar component of the carbon fibers, respectively.

In accordance with GB/T 1447-2005 [27], tensile tests were conducted on composite material specimens using a CMT5105 microcomputer-controlled electronic universal testing machine (100 kN) to determine the tensile strength and tensile modulus of the composite material.

The test specimens consisted of bone-shaped samples, measuring 180 mm in total length. The gauge length was specified as 50 ± 0.5 mm, with the distance between grips set at 115 ± 5 mm. The parallel section at the center had a width of 10 ± 0.5 mm, while the width at the ends was 20 ± 0.5 mm. The thickness of the specimens was maintained at 1.6 ± 0.1 mm. The tensile testing was conducted at a strain rate of 1 mm/min, and all reported values correspond to the mean results obtained from five individual specimens. All error bars in our manuscript represent the standard deviation (SD) calculated from multiple independent specimen tests. Tensile strength (MPa) is calculated using Equation (3), the specimen elongation at break is calculated using Equation (4), and the tensile modulus is calculated using Equation (5).

(3) $$\sigma_{t}=\frac{F}{b\cdot d}$$

where $\sigma_{t}$ is the tensile strength (MPa), *F* is the yield load (N), and *b* and *d* are the width and thickness of the specimen (mm), respectively.

(4) $$\varepsilon_{t}=\frac{∆L_{b}}{L_{0}}$$

where $\varepsilon_{t}$ is the elongation at fracture (%), $∆L_{b}$ is the extension of the gauge length at tensile fracture (mm), and $L_{0}$ is the original gauge length (mm).

(5) $$E_{t}=\frac{L_{0}\cdot ∆F}{b\cdot d\cdot ∆L}$$

where $E_{t}$ is the tensile modulus (MPa), ∆F is the load increment (N) within the linear-elastic region of the load–displacement curve, and ∆L represents the corresponding elongation of gauge length $L_{0}$.

In accordance with GB/T 1449-2005 [28], three-point bending tests were conducted on composite material specimens using a CMT5105 microcomputer-controlled electronic universal testing machine (100 kN, Shanghai, China). The specimens’ dimensions were 80 mm by 15 mm by 1.6 mm. The support span length was set to 25.6 mm, and testing was conducted at a speed of 2 mm per minute. The reported results represent the average values obtained from five individual test specimens. Flexural strength is calculated using Equation (6), and flexural modulus is calculated using Equation (7).

(6) $$\sigma_{f}=\frac{3P\cdot l}{2b\cdot h^{2}}[1+4{(S/l}^{2})]$$

where $\sigma_{f}$, *P*, *l*, *b*, *h*, and *S* represent the flexural strength (MPa), breaking load (N), span length (mm), specimen width (mm), specimen thickness (mm), and deflection at the midpoint of the span (mm), respectively.

(7) $$E_{f}=\frac{l^{3}\cdot ∆P}{4b\cdot h^{3}\cdot ∆S}$$

where $E_{f}$ is the flexural modulus (MPa), ∆P is the load increment (N) within the linear-elastic region of the load–deflection curve, and ∆S is the corresponding deflection increment (mm) at the mid-span.

According to ASTM D2344 [29], the ILSS of carbon-fiber composites was determined using the three-point bending method on a CMT5105 microcomputer-controlled electronic universal testing machine (100 kN). The specimen dimensions were 12 mm × 4 mm × 2 mm, with a test speed of 2 mm/min and a span of 8 mm. Testing is conducted at room temperature. The reported results represent the average values obtained from five individual test specimens. ILSS is determined using Equation (8):

(8) $$ILSS=\frac{3P_{b}}{4bh}$$

where $P_{b}$ is the maximum load at fracture (N), and b and h are the width and thickness of the specimen (mm), respectively.

The IFSS between carbon fibers and epoxy resin was evaluated utilizing the micro-drop debonding test. Carbon-fiber monofilaments were secured at both ends of a rectangular metal frame using high-temperature adhesive. A mixture of epoxy resin and curing agent was prepared at a mass ratio of 4:1. The mixture was carefully deposited onto an individual fiber filament to create a micro-droplet with an approximate diameter of 40 μm, which was subsequently cured at 100 °C for 30 min. A microbond test was then conducted by debonding the fiber at a constant speed of 1 mm/min. The IFSS was determined by utilizing the maximum load observed during the fiber pull-out test, as described by Equation (9) as follows.

(9) $$IFSS=\frac{F_{max}}{\pi dl}$$

where $F_{max}$ is the peak load (mN), d is the diameter of the carbon fiber (μm), and l is the diameter of the resin micro-droplet (μm).

## 3. Results and Discussion

### 3.1. Carbon-Fiber Surface Morphology Analysis

**Figure 2:**
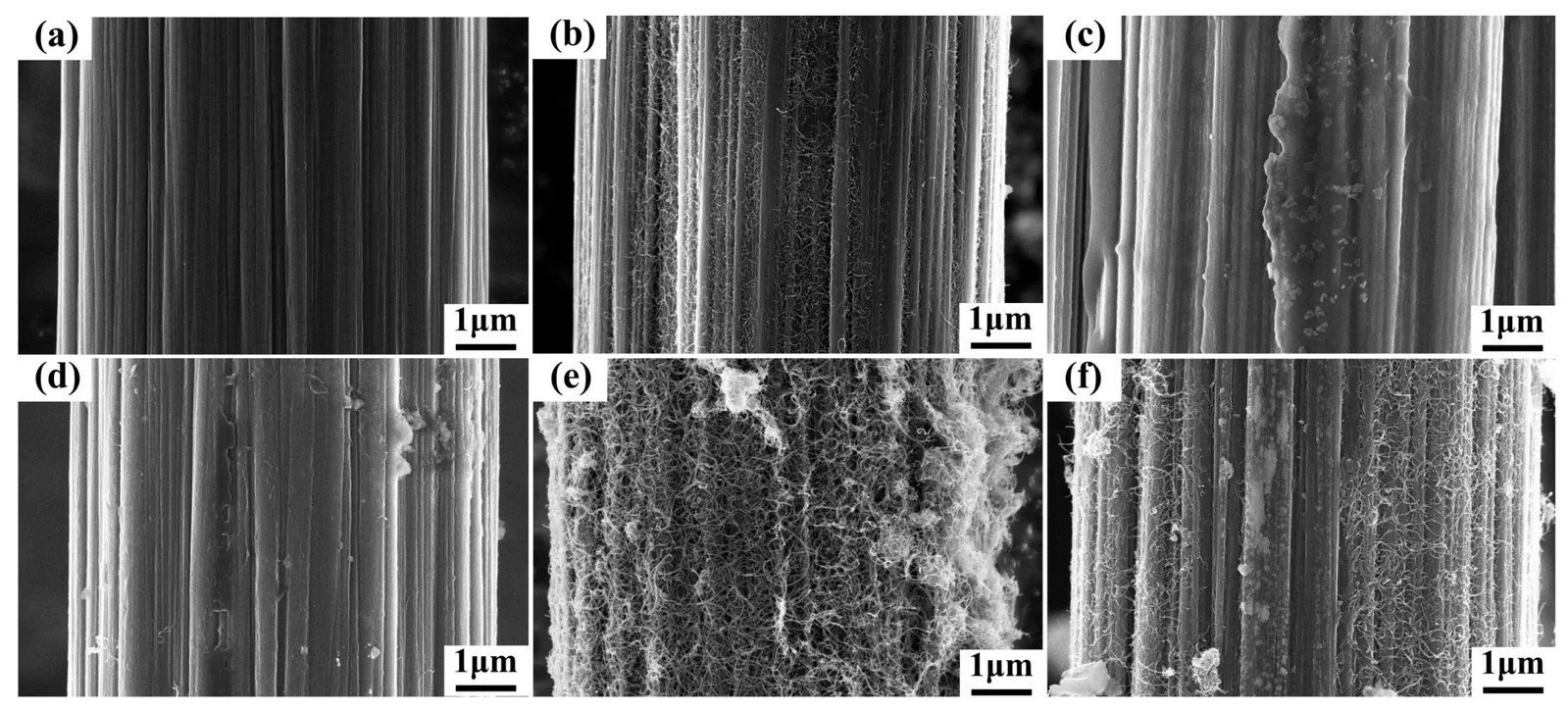
Surface morphologies of (**a**) CF, (**b**) CF-0.1%CNT, (**c**) CF-PDA-CNF, (**d**) CF-0.05%CNT-PDA-CNF, (**e**) CF-0.1%CNT-PDA-CNF, and (**f**) CF-0.2%CNT-PDA-CNF.

The morphological differences of carbon fibers obtained through various surface modification methods were observed by SEM. Figure 2a illustrates the surface morphology of the desized CFs, revealing grooves of differing depths aligned parallel to the fibers’ axial direction. The surface exhibits a smooth texture without any significant deposits or protruding features. Figure 2b shows that CNTs are uniformly attached to the surface of CF-0.1%CNT, with increased surface roughness compared to the desized CFs. However, the presence of narrow grooves on the carbon-fiber surface remains evident following the grafting of CNTs. Figure 2c shows the surface morphology of CF-PDA-CNF. Without the presence of CNTs, it is difficult for a three-dimensional network layer to form on the fiber surface. The fiber surface is coated with a discrete PDA layer, characterized by the presence of several granular protrusions. Mass fractions of CF-xCNT-PDA-CNF (x = 0.05%, 0.1%, 0.2%) were obtained by varying the content of CNTs, as shown in Figure 2d–f. CNFs uniformly distributed on the surface of CF-0.1%CNT-PDA-CNF exhibited a fluffy albeit rough overall state. Intertwining with the carbon-fiber surface, CNFs formed a complex “three-dimensional nanospider web” structure and created new layers. This modification increased the number of mechanical interlocks between the carbon fibers and the matrix, thereby improving the interfacial adhesion between them. Figure 2d,f exhibit fewer CNFs nanowires adhering to the surface. This occurs because an insufficient number of CNTs struggles to form a continuous reinforcing network on the CF surface, while an excessive number of CNTs tends to agglomerate and detach, thereby hindering CNFs from adhering to the carbon-fiber surface.

**Figure 3:**
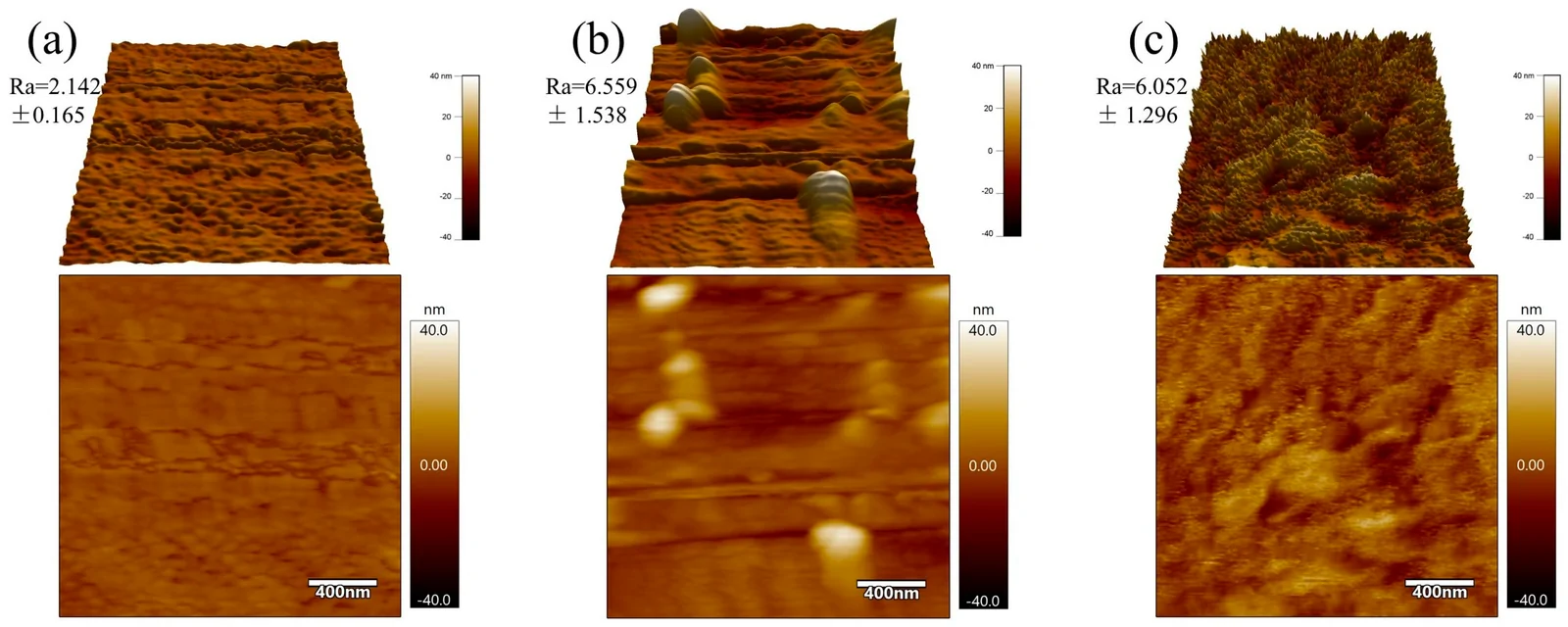
AFM images of (**a**) CF, (**b**) CF-0.1%CNT, and (**c**) CF-0.1%CNT-PDA-CNF. The quoted Ra values in the figure are given as mean ± standard deviation (mean ± SD), which are calculated from five independently scanned regions of each sample.

The three-dimensional morphology and roughness evolution of carbon fibers were characterized by AFM. The data plotted in the figure represent the mean ± standard deviation calculated from several scanned regions, and only average values have been used when describing numerical variations in the following text. As shown in Figure 3a, the desized CF surface appears relatively smooth, exhibiting shallow grooves along the fiber axis without discernible particulate attachments. The corresponding surface roughness (Ra) was measured to be 2.142 nm. Figure 3b shows that CNT grafting resulted in a significantly higher surface roughness (Ra = 6.559 nm) for the carbon fibers, with the morphology characterized by randomly distributed protruding “hill” structures along the grooves. Figure 3c shows that the deposition of PDA and CNFs partially filled the surface grooves, resulting in a reduced Ra of 6.052 nm. Collectively, the introduced CNTs, PDA, and CNFs effectively increased the overall fiber surface roughness, which contributed to enhanced mechanical interlocking with the epoxy matrix.

### 3.2. CF Surface Composition Analysis

**Figure 4:**
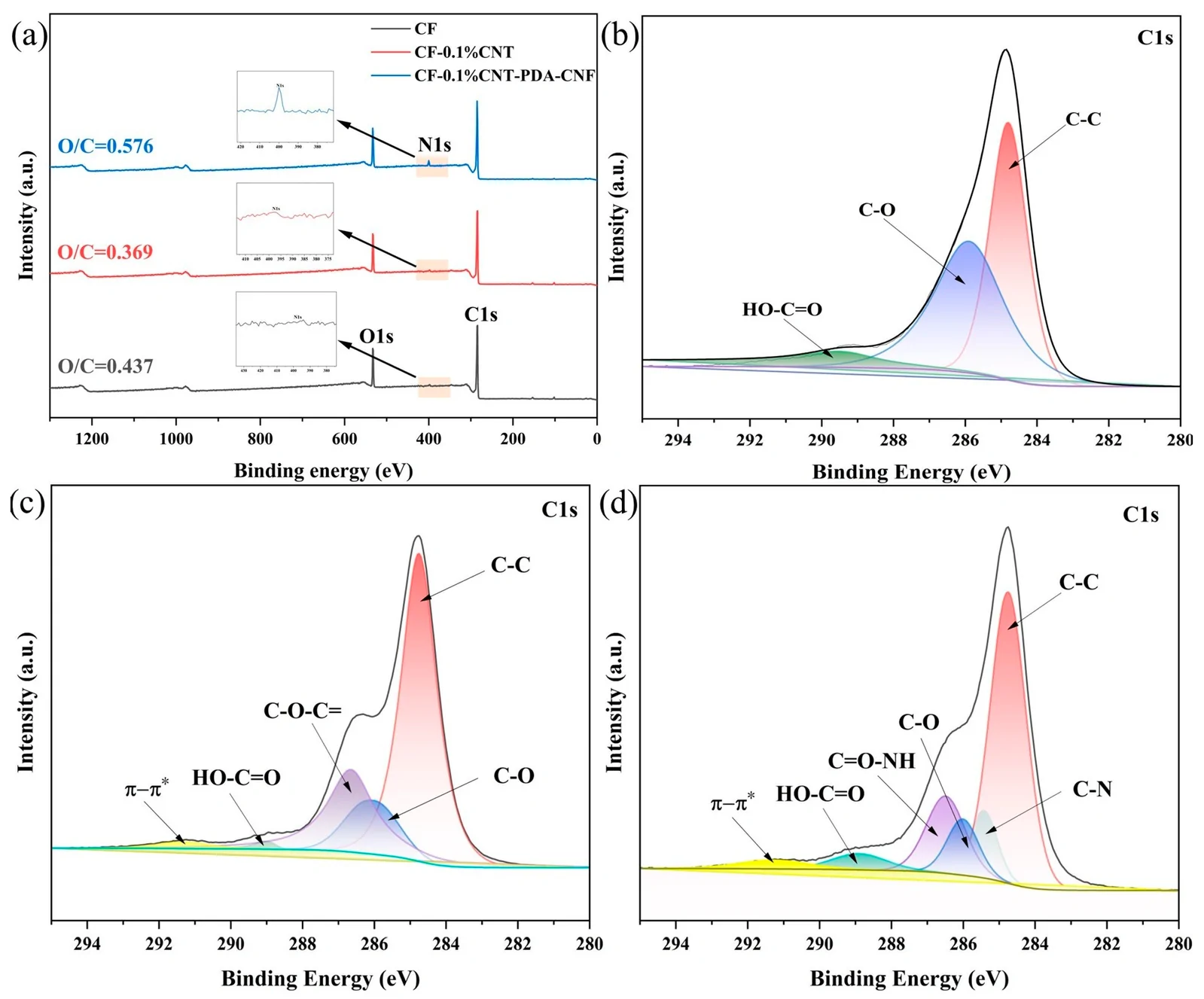
(**a**) Full XPS spectra and high-resolution C1s scan spectra of (**b**) CF, (**c**) CF-0.1%CNT, (**d**) CF-0.1%CNT-PDA-CNF.

Chemical bonding establishes more stable and robust interfacial connections through atomic-level chemical interactions. The surface chemical composition of various carbon-fiber specimens was analyzed utilizing XPS, as illustrated in Figure 4a. The comprehensive spectra of the carbon-fiber samples reveal prominent C1s and O1s peaks, signifying the presence of carbon and oxygen elements within the samples. The CF-0.1%CNT sample exhibited characteristics similar to the CF sample, with the carbon content increasing from 69.58% to 73.04% and the oxygen content decreasing from 30.42% to 26.96% (Table 1). This indicates that CNTs were successfully grafted onto the CF surface via a dehydration–condensation reaction. The CF-0.1%CNT-PDA-CNF sample exhibited N1s peaks in addition to C1s and O1s peaks (Table 1, nitrogen content: 4.11%), indicating successful grafting of PDA onto the CF surface. The carbon concentration declined from 73.04% to 60.81%, whereas the oxygen concentration rose from 26.96% to 35.08%. The surfaces of the CNFs are abundant in carboxyl and hydroxyl functional groups, thereby confirming the effective integration of CNFs onto the carbon fibers.

Chemical components were analyzed in greater detail using C1s high-resolution spectra, as shown in Figure 4b–d. The CF sample (Figure 4b) exhibits C-C bonds (~284.8 eV), C-O bonds (~285.5 eV), and HO-C=O bonds (~289.5 eV) [30,31]. As illustrated in Figure 4c, the C-C bond remains the dominant peak in the CF-0.1%CNT sample. Compared to the CF sample, an additional peak emerges at 286.6 eV, which is attributed to the carbonyl oxygen atom (C-O-C=) present in the anhydride [32]. This further confirms the dehydration reaction between carbon fiber and the carboxyl groups of CNTs. Figure 4d illustrates that the CF-0.1%CNT-PDA-CNF sample contains C-N bonds (~286.0 eV), which are attributed to the interaction between the amino groups present in PDA and the carboxyl groups on the CNTs and CNFs, in addition to the C-C, C-O, and HO-C=O bonds. This suggests that the surface of CF-CNT has been effectively functionalized with PDA. Compared to the CF-0.1%CNT sample, the presence of a C=O-NH bond (~286.5 eV) can be observed, which is attributed to the formation of an amide linkage resulting from the reaction between the carboxyl groups of CNFs and the amino groups of PDA. The appearance of π-π* satellite peaks (~290–292 eV) results from interactions between the aromatic rings of PDA and the sidewalls of CNTs [33].

### 3.3. CF Surface Defect Analysis

**Figure 5:**
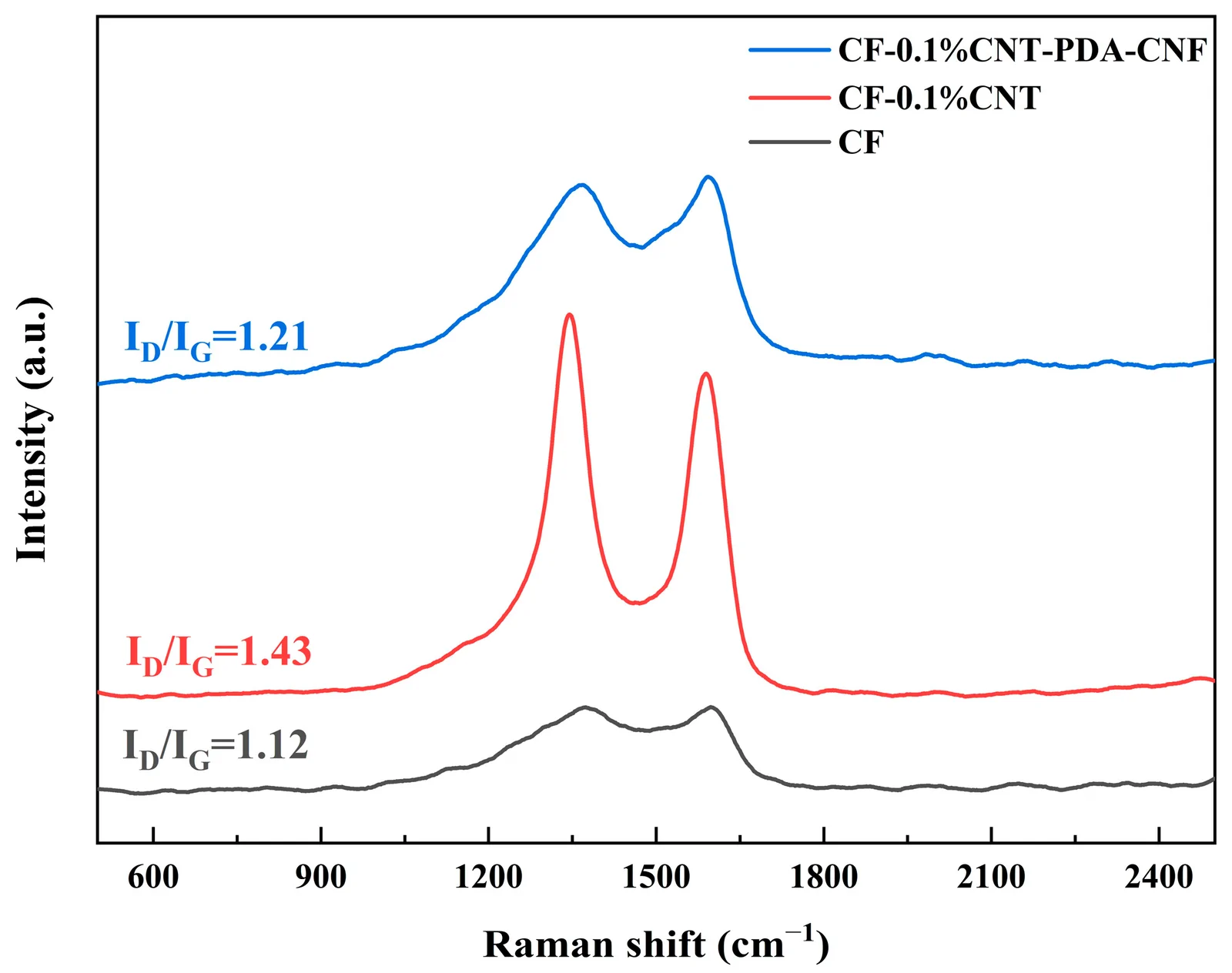
Raman spectroscopy analysis of carbon fibers treated with different modification methods.

Raman spectroscopy was employed to evaluate the structural defects of different modified carbon-fiber samples. The D peak (~1350 cm^−1^) of carbon fiber reflects defects or disordered carbon in its graphitic structure. The G peak, observed at approximately 1580 cm^−1^, is attributed to the well-ordered vibrational modes within the graphitic lattice structure [34]. As illustrated in Figure 5, the intensity of the D peak exhibits an increase following the grafting of CNTs (CF-0.1%CNT). This enhancement is dominantly attributed to intrinsic structural defects originating from the grafted CNTs themselves. Although the 950 °C high-temperature treatment under argon protection may induce minor surface defects on the carbon-fiber substrate, thermal effects are not the major driving factor for the increased D peak intensity and (I_D_/I_G_) ratio in CF-0.1%CNT. The G peak shifts toward higher wavenumbers (~1585–1600 cm^−1^) and exhibits a sharper peak shape, indicating a significant level of graphitization in the CNTs and the preservation of the structural integrity of their walls. The I_D_/I_G_ ratio serves as an indicator of the extent of graphitization in carbon-based materials [35]. After coating CF-0.1%CNT with PDA and CNFs (CF-0.1%CNT-PDA-CNF), both the D peak and G peak signals corresponding to CNTs were weakened. This is attributed to CNFs bonding with CNTs and PDA through covalent and hydrogen bonds. Their own amorphous region signals partially mask the D peak of CNTs, leading to peak broadening. Simultaneously, the physical coverage of PDA on CNFs reduces the signal intensity of the G peak of CNTs. Additionally, the covalent bond interactions formed between PDA and CNTs cause the G peak to shift further toward higher wavenumbers (~1600 cm^−1^).

### 3.4. Analysis of Carbon-Fiber Surface Wettability

**Figure 6:**
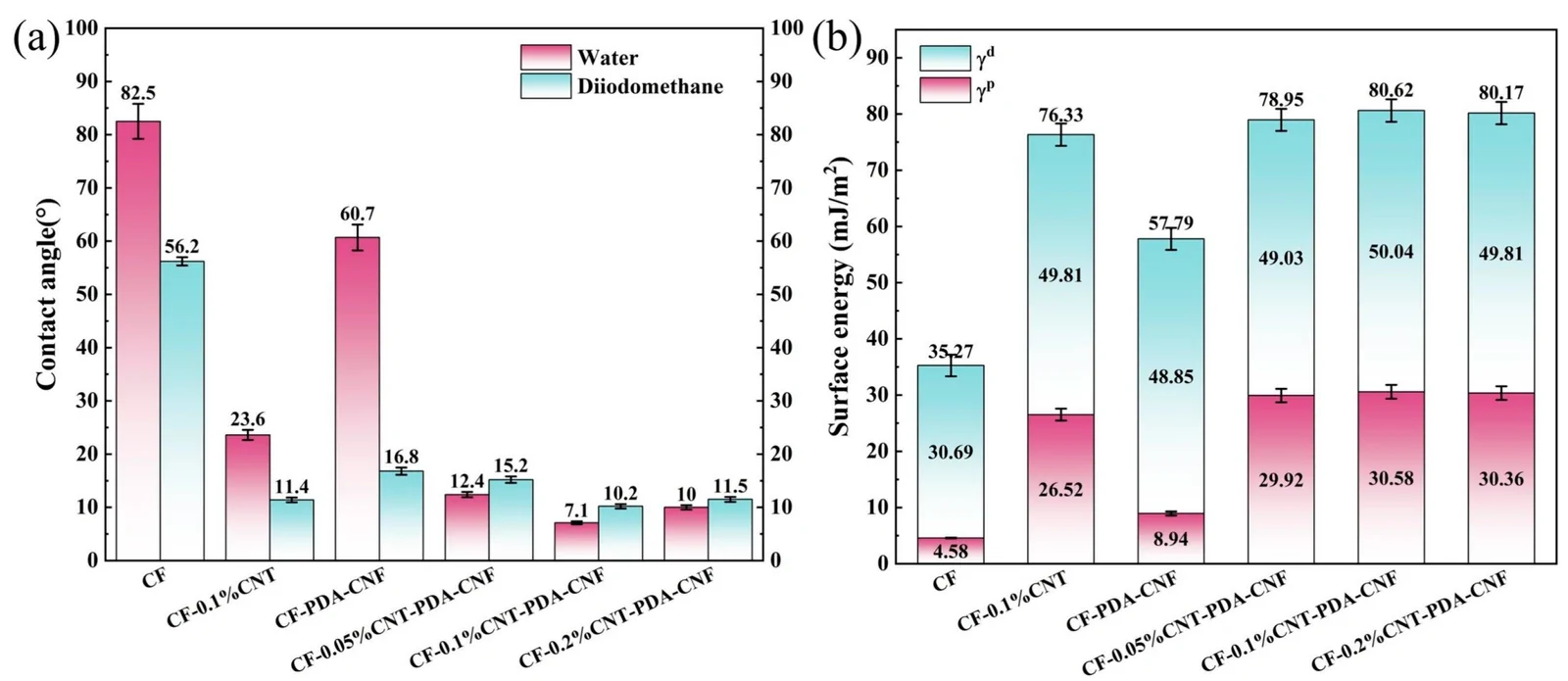
Contact angles (**a**) and surface energies (**b**) of carbon fibers subjected to various modification methods. Error bars represent standard deviation obtained from *n* = 10 independent specimens.

Figure 6a shows the measured contact angle values of carbon fibers when exposed to water and diiodomethane. Data in the figures are presented as mean ± standard deviation obtained from multiple independent specimens; only average values have been adopted to describe numerical variations in the following text. The contact angles of CF, CF-0.1%CNT, CF-PDA-CNF, CF-0.05%CNT-PDA-CNF, CF-0.1%CNT-PDA-CNF, and CF-0.2%CNT-PDA-CNF with water and diiodomethane were 82.5°, 23.6°, 60.7°, 12.4°, 7.1°, and 10° and 56.2°, 11.4°, 16.8°, 15.2°, 10.2°, and 11.5°, respectively. All contact angle values were collected at a fixed delay of 1 s after droplet deposition. It should be noted that the apparent wettability of rough nanostructured surfaces arises from the interplay between surface chemical composition and nano-scale topography rather than being governed solely by roughness or chemical groups. Increased surface roughness does not unconditionally improve hydrophilicity, as reported in the previous literature [36,37]. Figure 6b illustrates the trend in surface energy. After CNT grafting, both surface roughness and the contents of polar carboxyl groups were elevated, which jointly contribute to the pronounced reduction in the contact angle of CF-0.1%CNT. The dispersion component increased from 30.69 mJ/m^2^ to 49.81 mJ/m^2^. Abundant carboxyl groups on CNT surfaces raised the polar component of the CF-0.1%CNT sample from 4.58 mJ/m^2^ to 26.52 mJ/m^2^. However, the contact angle of the CF-PDA-CNF sample did not exhibit a notable reduction, which can be attributed to the PDA coating filling the pre-existing grooves on the fiber surface. The CF-0.1%CNT-PDA-CNF sample demonstrated the minimum contact angle alongside the maximum surface energy, characterized by polar and dispersive components measuring 50.04 mJ/m^2^ and 30.58 mJ/m^2^, respectively. The surface energy of unmodified CF was measured to be 35.27 mJ/m^2^, which aligns with the values documented in previous studies [38,39]. The surface energies of CF-0.1%CNT, CF-PDA-CNF, CF-0.05%CNT-PDA-CNF, CF-0.1%CNT-PDA-CNF, and CF-0.2%CNT-PDA-CNF were 76.33 mJ/m^2^, 57.79 mJ/m^2^, 78.95 mJ/m^2^, 80.62 mJ/m^2^, and 80.17 mJ/m^2^, respectively. CF-0.1%CNT-PDA-CNF exhibited optimal surface properties, with its surface energy increased by 128.60% compared to CF. The increase in polar component stems from polar functional groups provided by CNTs, PDA, and CNFs, such as -OH, -NH_2_, and -COOH [40,41]. The “three-dimensional nanospider web” network remodels the surface topography of carbon fibers and increases surface roughness (Ra increases from 2.142 nm to 6.559 nm). Nevertheless, a higher surface roughness does not necessarily lead to enhanced hydrophilicity, as the surface topography can either amplify or counteract the effects of polar functional groups [42]. Consequently, the ultimate improvement in wettability is the combined result of coupled surface chemical and morphological effects.

### 3.5. Analysis of Mechanical Properties of CF/EP Composite Materials

**Figure 7:**
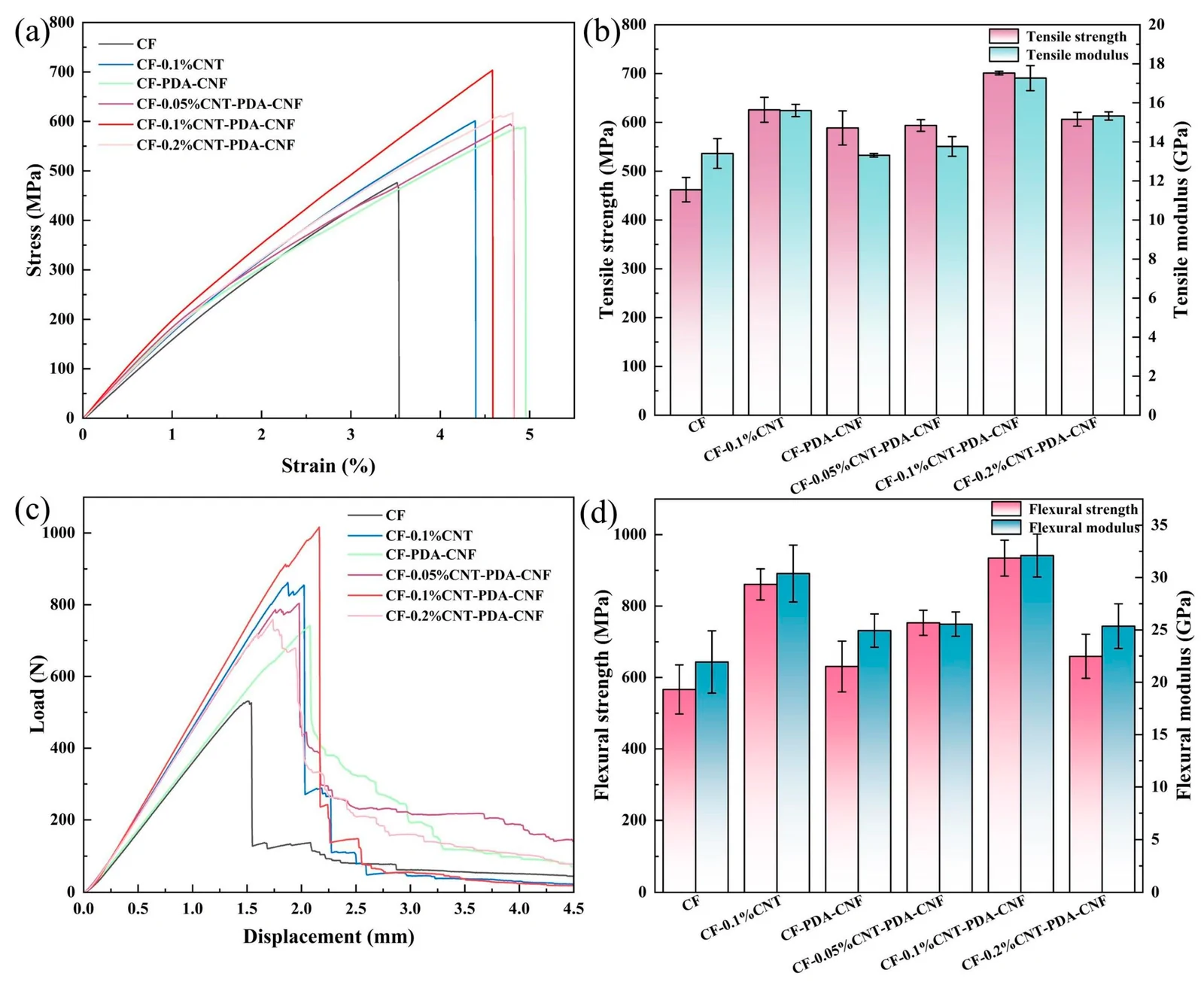
(**a**) Tensile stress–strain curve, (**b**) tensile strength and tensile modulus, (**c**) bending load–displacement curve, and (**d**) bending strength and bending modulus of CF/EP composite materials. Error bars represent standard deviation obtained from *n* = 5 independent specimens.

Tensile strength, flexural strength, ILSS, and IFSS correspond to a composite material’s resistance capabilities under different loading conditions. These properties directly determine whether the composite can withstand specific loads without failure in practical applications, making them key parameters for evaluating its practical application potential [43]. The tensile test data shown in Figure 7a,b clearly demonstrate the differences in key performance indicators, such as tensile strength, tensile modulus, and elongation at break, for the composite materials. The tensile properties of CF-xCNT-PDA-CNF-reinforced composites initially enhance and subsequently decline as the CNT content increases. The CF-0.1%CNT-PDA-CNF composite exhibited the highest tensile strength of 701.08 MPa, with a tensile modulus of 17.26 GPa. Compared to the unmodified CF-reinforced composite (462.02 MPa, 13.41 GPa), these measurements correspond to enhancements of 51.7% and 28.7%. The fracture elongation increased from 3.54% to 4.58%. According to the CNT interface optimization concentration threshold theory [44], when the concentration of CNTs decreases below a critical threshold, the dispersed CNTs are unable to establish a continuous reinforcing network within the matrix. Conversely, when the concentration is excessively high, stress concentration points arising from local agglomeration significantly degrade the material’s overall performance. As a result of the π-π interactions occurring between PDA and the carbon fibers, a compact PDA layer is established on the surface of the carbon fibers. This coating not only improves interfacial adhesion between the fibers and the matrix but also embeds CNTs and CNFs into the PDA layer, forming a three-dimensional network structure. This structure facilitates load transfer between carbon fibers and the resin, thereby augmenting the tensile performance of the composite material.

Figure 7c,d analyze the bending stress–strain response and the variation patterns of mechanical properties for different composite materials. The flexural properties of all modified CF composites were enhanced. The CF-0.1%CNT-PDA-CNF composite exhibited a pronounced strain-hardening phase prior to fracture, indicating its superior energy dissipation capacity. Compared to the CF-reinforced composite, the CF-0.1%CNT-PDA-CNF composite exhibited superior ductility, with flexural strength and flexural modulus increasing to 934.41 MPa and 32.11 GPa, representing improvements of 64.98% and 26.8%, respectively. This is attributable to the “three-dimensional nanospider web” structure formed by CNTs, PDA, and CNFs. The nanonetwork layer of this structure can undergo a certain degree of deformation, slip, and fracture, facilitating more efficient stress transfer from the matrix to the fibers. Consequently, the flexural strength and flexural modulus of the composite materials are improved.

**Figure 8:**
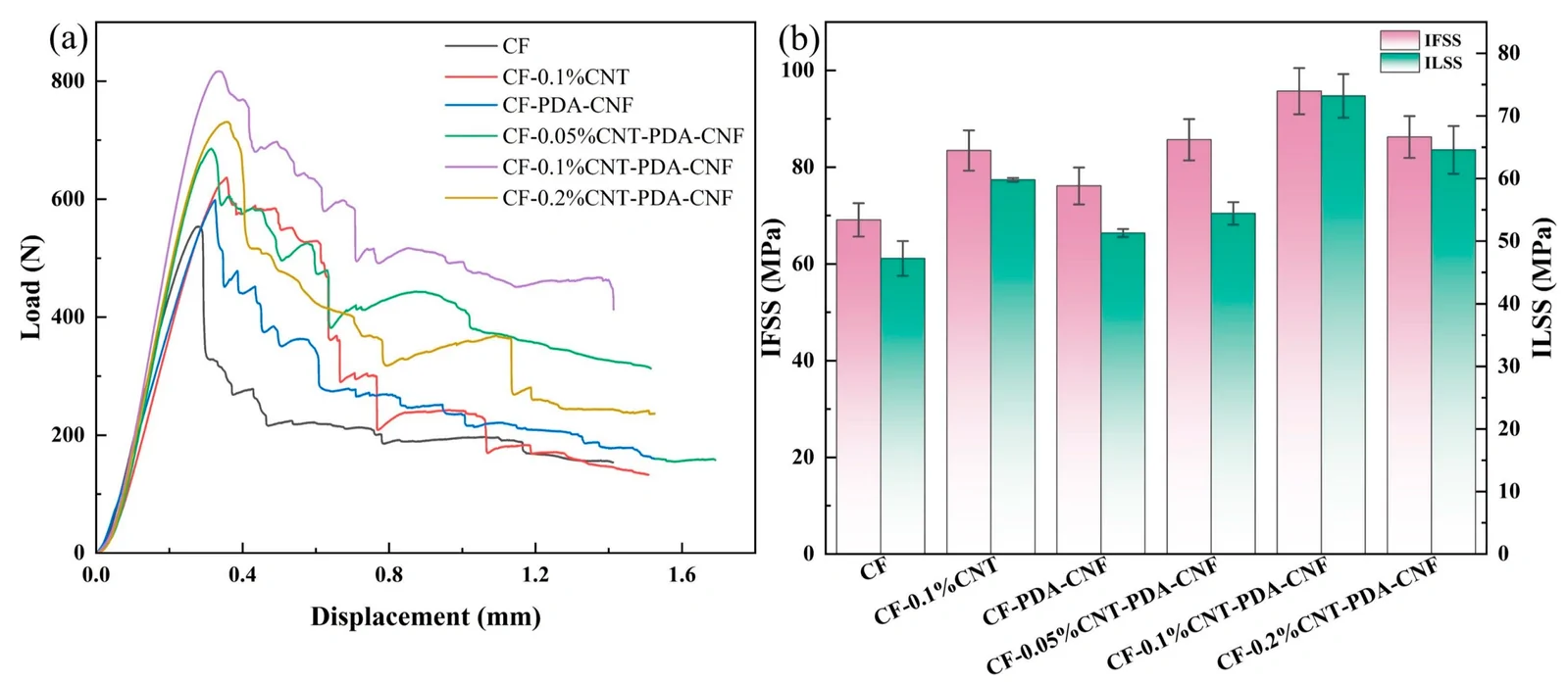
Interface properties of CF/EP composite materials: (**a**) interlaminar shear load–displacement curve; (**b**) ILSS and IFSS. Error bars represent standard deviation obtained from *n* = 5 independent specimens.

ILSS is obtained from short-beam shear testing on macroscopic composite laminates. Figure 8a displays the interlaminar shear strength displacement–load curves for the composite materials, reflecting the entire process from elastic deformation, crack initiation, and delamination propagation through to macroscopic failure. Following surface modification, all composites showed an increase in ILSS. The composite based on CF-0.1%CNT-PDA-CNF demonstrated the highest initial slope in the elastic region, as shown in its stress–strain curve, indicating superior shear stiffness. The area under the load–displacement curve can be directly used to quantify the ability to dissipate interfacial energy, indicating that this composite material can absorb more energy during the interfacial damage process. ILSS is shown in Figure 8b. The CF/EP composite exhibited the lowest ILSS (47.24 MPa) and experiences delamination failure at lower loads, indicating weak fiber–matrix bonding. The ILSS of the CF-0.1%CNT/EP composite exhibited a slight improvement, reaching 59.8 MPa. This enhancement is primarily ascribed to the elevated modulus of the CNTs and the increased specific surface area of the carbon fibers. The ILSS values of the CF-PDA-CNF-, CF-0.05%CNT-PDA-CNF-, and CF-0.2%CNT-PDA-CNF-reinforced composites were 51.29 MPa, 54.44 MPa, and 64.57 MPa, respectively. In comparison, the CF-0.1%CNT-PDA-CNF composite achieved the highest ILSS of 73.19 MPa, representing an improvement of 54.93% relative to the baseline CF/EP composite.

IFSS was adopted to evaluate the interfacial-related performance of composites. IFSS, derived from the micro-drop debonding test, quantifies the intrinsic shear-resisting capability of the single fiber–matrix interphase at the micro-scale and serves as a direct indicator of fiber–matrix bonding quality. As illustrated in Figure 8b, the IFSS of the CF/EP composite material is 69.12 MPa. The introduction of CNTs (CF-0.1%CNT) significantly increased the fiber surface roughness, forming greater mechanical interlocking with the resin and elevating the IFSS to 84.45 MPa. The IFSS values of the CF-PDA-CNF-, CF-0.05%CNT-PDA-CNF-, CF-0.1%CNT-PDA-CNF-, and CF-0.2%CNT-PDA-CNF-reinforced composites were 76.22 MPa, 85.67 MPa, 95.71 MPa, and 86.24 MPa, respectively. The CF-0.1%CNT-PDA-CNF-reinforced composite exhibited the highest IFSS, representing a 38.39% improvement over the CF/EP composites. This indicates that the nanostructured layer can withstand considerable shear loading without large-scale delamination from the fiber surface. Within this structure, the sequentially decreasing moduli of CNTs, PDA, and CNFs, coupled with the partial embedding of CNFs into the epoxy matrix, establish a continuous modulus transition from the fibers to the resin. This design effectively alleviates the stress concentration typically induced by an abrupt modulus mismatch at the interface. The simultaneous improvement in both micro-scale IFSS and macro-scale ILSS mutually validates that our nanonetwork interphase modification effectively enhances interfacial bonding, which further translates into improved macroscopic composite performance.

### 3.6. Fracture Morphology Analysis of CF/EP Composite Materials

**Figure 9:**
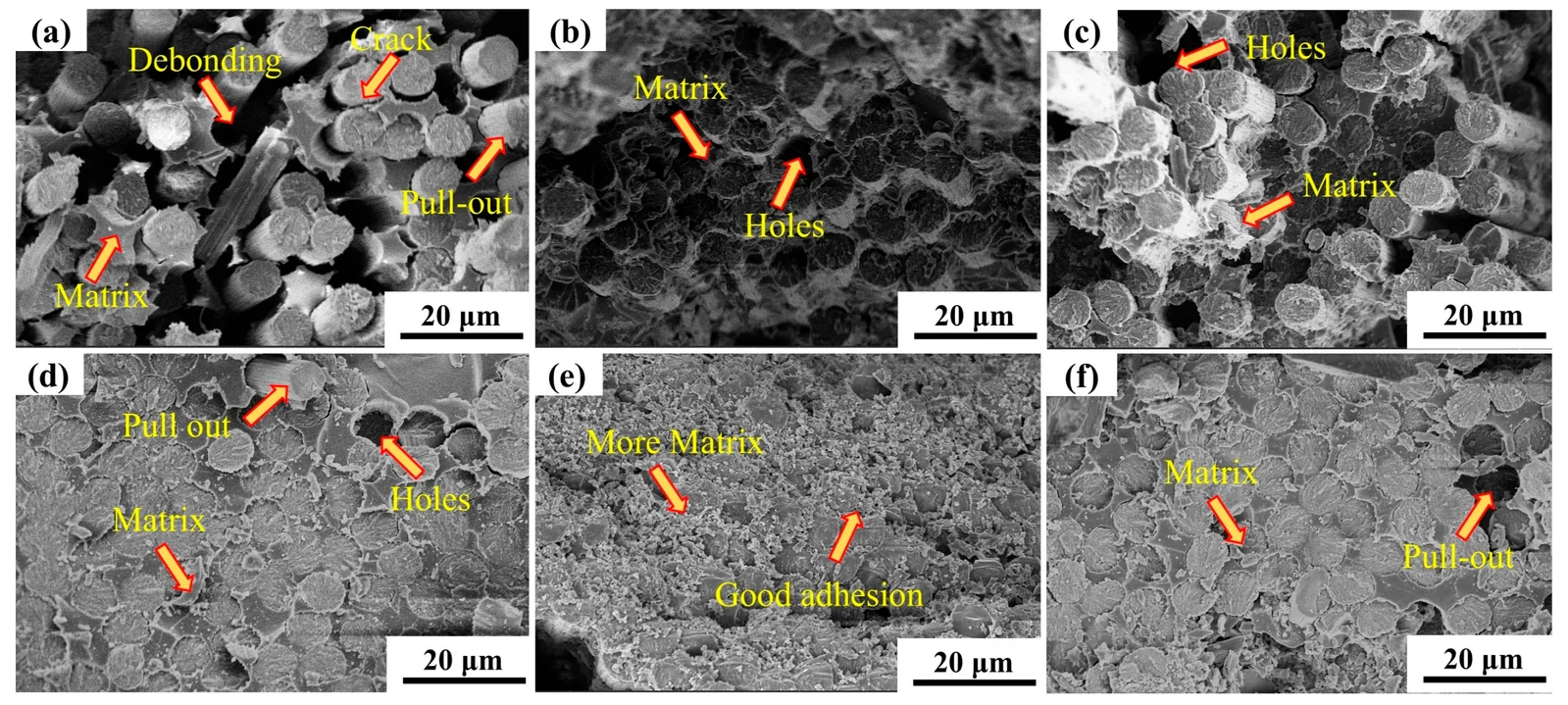
Tensile fracture cross-sectional morphologies of CF/EP composites: (**a**) CF, (**b**) CF-0.1%CNT, (**c**) CF-PDA-CNF, (**d**) CF-0.05%CNT-PDA-CNF, (**e**) CF-0.1%CNT-PDA-CNF, and (**f**) CF-0.2%CNT-PDA-CNF.

**Figure 10:**
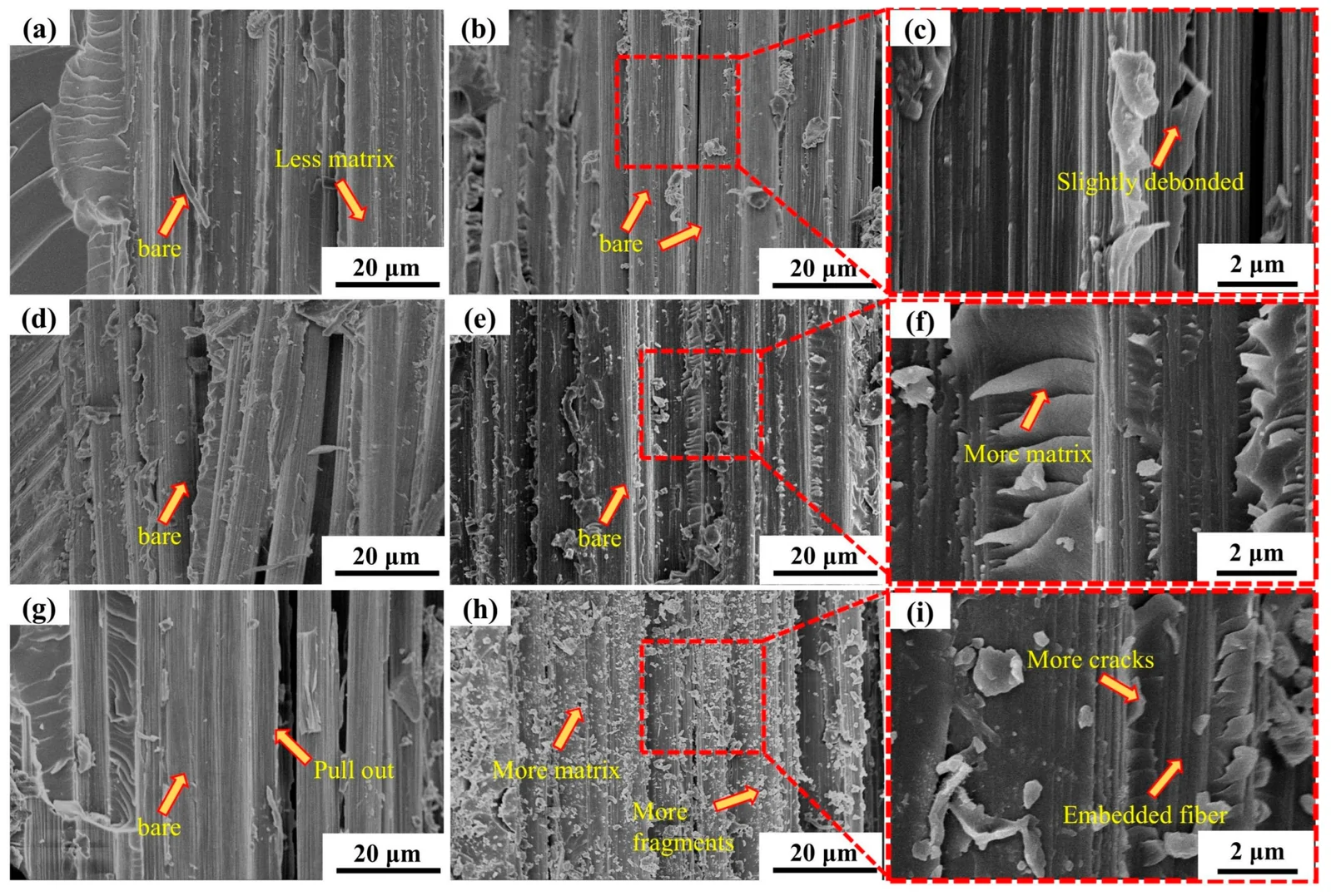
Longitudinal fracture cross-sectional morphologies of CF/EP composites: (**a**) CF-PDA-CNF, (**b**,**c**) CF, (**d**) CF-0.05%CNT-PDA-CNF, (**e**,**f**) CF-0.1%CNT, (**g**) CF-0.2%CNT-PDA-CNF, and (**h**,**i**) CF-0.1%CNT-PDA-CNF.

To elucidate the failure behavior of composite materials, SEM was employed to examine the cross-sections and longitudinal sections of specimens after tensile testing. It should be noted that these are post-fracture observations, which provide indirect evidence for interfacial failure behavior rather than capturing real-time interfacial evolution under applied loads. The findings are presented in Figure 9 and Figure 10. Extensive fiber pull-out can be observed on the fracture surfaces of the CF/EP composites, as illustrated in Figure 9a and Figure 10b,c. The pulled-out fibers display smooth surfaces, suggesting that failure occurred primarily via adhesive debonding at the fiber–matrix interface. The matrix region contains a high density of voids, which serve as stress concentrators that promote crack propagation along the interface. This phenomenon indicates inadequate interfacial adhesion, attributable to the absence of chemical bonding between the fibers and the resin. Furthermore, stress distribution irregularities caused by the modulus mismatch exacerbate these defects, making the interface the primary failure point [19]. The integration of CF-0.1%CNT markedly enhanced the interfacial characteristics of the composite material. As illustrated in Figure 9b and Figure 10e,f, the internal porosity was markedly reduced, although minor fiber pull-out still occurred. In the longitudinal cross-section of CF-0.1%CNT/EP, effective adhesion of the resin matrix to the fiber surface was evident. This indicates that CNTs enhance interfacial bonding primarily through mechanical interlocking, leading to a mixed failure mode involving both adhesive and cohesive failure [45].

For the CF-0.1%CNT-PDA-CNF/EP composite material (Figure 9e and Figure 10h,i), the fracture surface exhibits a comparatively smooth texture, characterized by a dense adhesion of carbon fibers to the resin matrix. Virtually no voids are observable in the cross-section. The carbon-fiber surface is covered by a continuous and complete resin layer, further confirming the presence of strong interactions dominated by chemical bonding at the interface [46]. Additionally, stress gradients between adjacent carbon fibers induce microcrack propagation paths. This demonstrates that the three-dimensional network synergistically constructed by CNFs, PDA, and CNTs can effectively transfer and dissipate stress. The fracture morphology suggests a transition in the failure mechanism from being governed primarily by the interface to being dominated by the matrix. This shift indicates that the bonding strength at the interface has exceeded the intrinsic strength of the matrix material. The interfacial debonding process is characterized by a mixed failure mode: adhesive failure at the fiber–matrix interface in some areas, alongside cohesive failure involving plastic deformation and fracture of the surrounding resin matrix in adjacent regions. The formation of this dual-mode fracture mechanism stems from the interfacial enhancement effect of CNT-PDA-CNF. CNT-PDA-CNF, featuring a “three-dimensional nanospider web” structure, optimizes stress transfer between the carbon fibers and the resin matrix through chemical bonding and modulus transition. This effectively regulates the energy dissipation pathways, ultimately enhancing the interfacial bonding strength.

### 3.7. Analysis of Interface Properties in CF/EP Composite Materials

**Figure 11:**
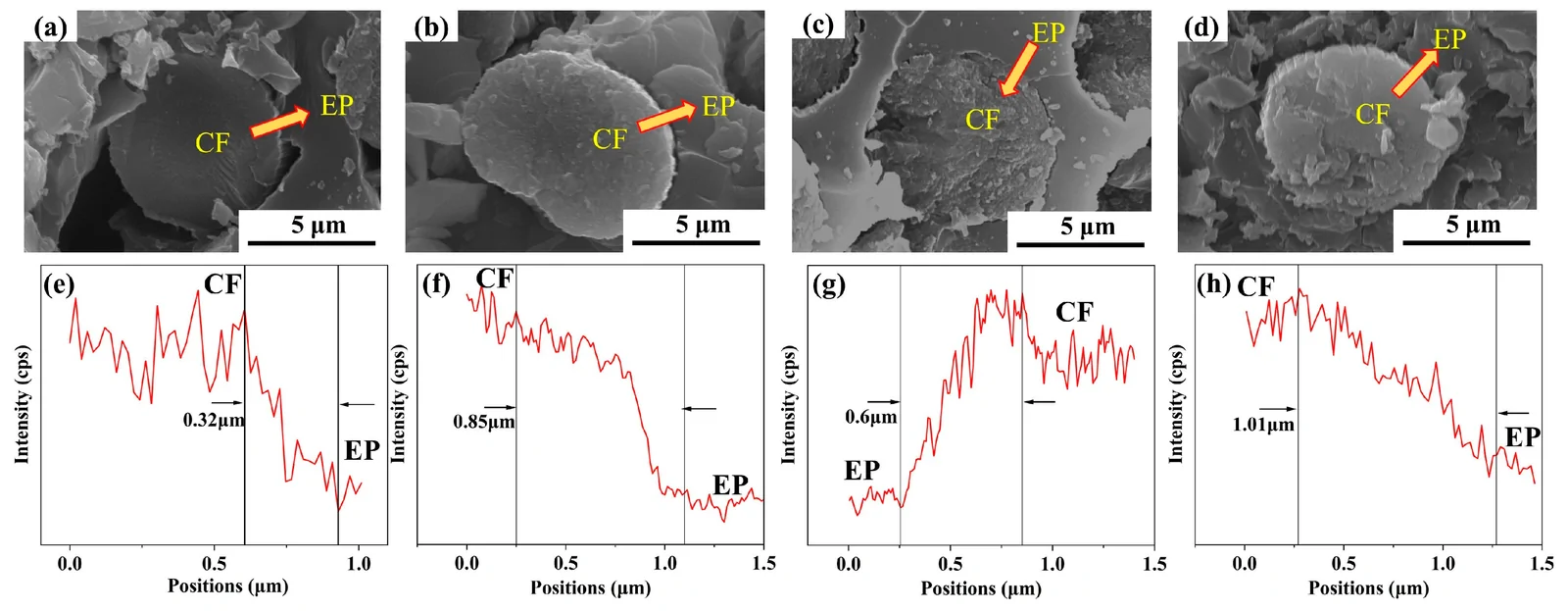
Interfacial morphologies and carbon element distributions of different CF/EP composites: (**a**,**e**) CF, (**b**,**f**) CF-0.1%CNT, (**c**,**g**) CF-PDA-CNF, and (**d**,**h**) CF-0.1%CNT-PDA-CNF.

EDS line scanning was performed to analyze the carbon-signal distribution and estimate the interphase thicknesses of different CF/EP composites [47]. It should be noted that these EDS line-scan measurements were obtained on rough, non-planar fracture surfaces. Raw signal intensities can be influenced by local surface inclination and geometrical effects; hence, these results provide only indirect qualitative evidence instead of strict quantitative elemental-concentration data. As shown in Figure 11, the CF/EP composite exhibited a sharp decline in carbon content from the fibers to the matrix, corresponding to a narrow interface thickness of only 0.32 μm. In contrast, after CNT grafting (CF-0.1%CNT), the interface broadened to 0.85 μm, with the carbon content exhibiting a gradual initial decrease followed by a sharp drop. With the subsequent grafting of PDA and CNFs (CF-0.1%CNT-PDA-CNF), the interfacial thickness further increased to 1.01 μm, accompanied by a smoother gradient in the carbon distribution profile. This thickened and graded interface results in more effective stress transfer and dispersion, thereby requiring greater energy to impede crack propagation and ultimately enhancing the interfacial bonding performance [12]. For the CF-PDA-CNF/EP composite without CNTs (Figure 11c,g), the interfacial thickness showed a slight increase, with a relatively steep curve slope.

**Figure 12:**
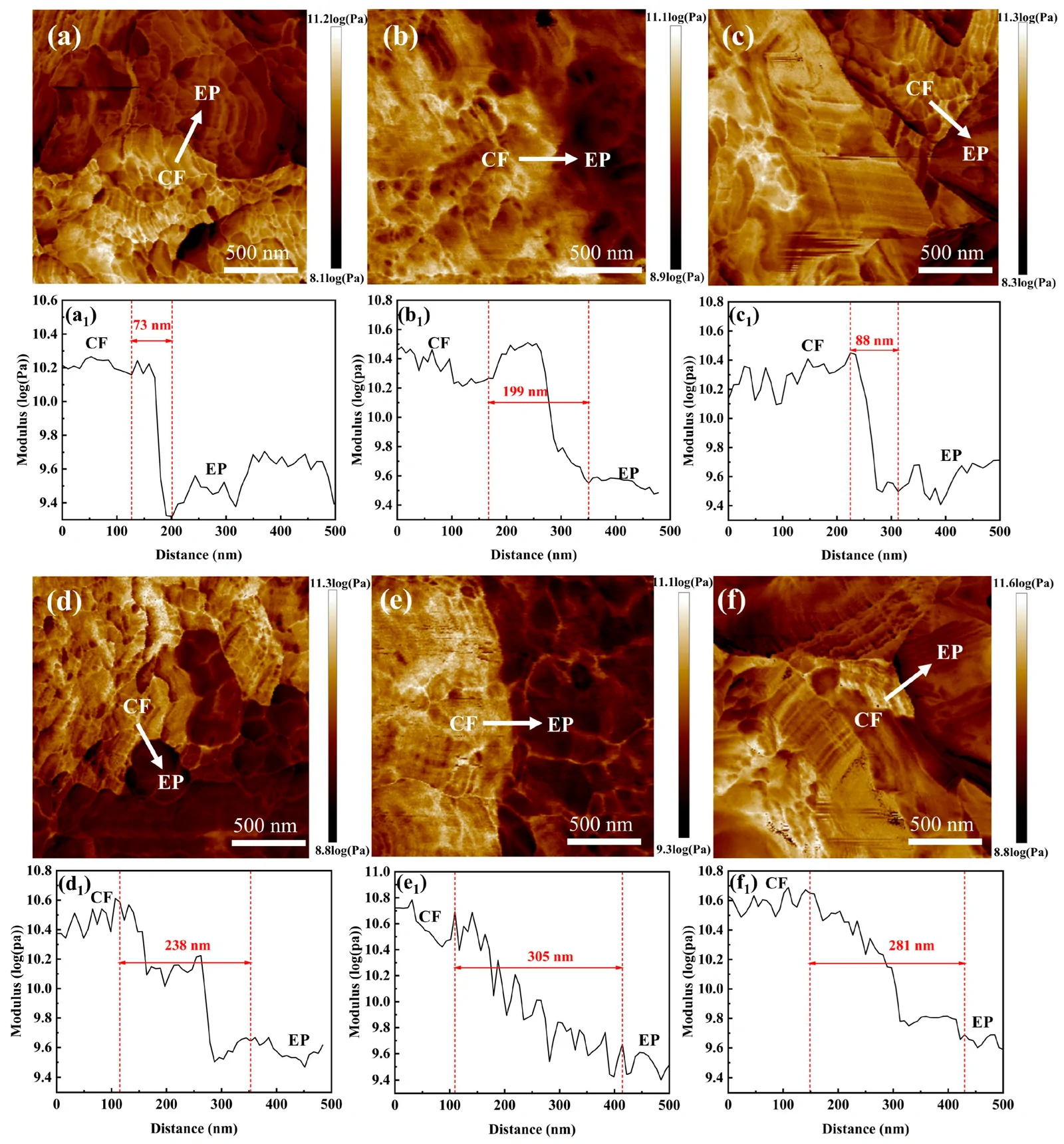
Distribution of elastic moduli at the interfaces of different CF/EP composites, with the corresponding elastic moduli line distributions along the arrow direction: (**a**,**a_1_**) CF, (**b**,**b_1_**) CF-0.1%CNT, (**c**,**c_1_**) CF-PDA-CNF, (**d**,**d_1_**) CF-0.05%CNT-PDA-CNF, (**e**,**e_1_**) CF-0.1%CNT-PDA-CNF, and (**f**,**f_1_**) CF-0.2%CNT-PDA-CNF.

AFM force–modulation (PF-QNM) curves were employed to characterize the modulus transition across the carbon-fiber-to-resin interphase. This measurement aims to probe the mechanical property distribution within the interphase region, which is central to validating our proposed gradient interphase reinforcement mechanism. The core role of a gradient modulus interphase is to alleviate the sharp stiffness mismatch between carbon fiber and epoxy resin. Its continuously varying modulus enables smooth stress transfer, relieves local stress concentration, and consequently improves composite interfacial adhesion. As presented in Figure 12, the modulus map, indicated by color variations, visually delineates the interfacial layer between the fiber and resin. The modulus variation curve clearly reflects the modulus change from carbon fibers to the resin [48,49]. In comparison to CF/EP composites, the interfacial modulus curve of CF-0.1%CNT/EP composites exhibited a small plateau, indicating that the incorporation of CNTs increased the modulus. In contrast to the CF-PDA-CNF/EP composite, where no distinct modulus transition layer is discernible at the interface, the CF-xCNT-PDA-CNF/EP composites (x = 0.05%, 0.1%, 0.2%) exhibited a pronounced interfacial transition zone. The thickness of this zone shows a non-monotonic dependence on CNTs content, increasing initially before decreasing at higher loadings. Among these samples, CF-0.1%CNT-PDA-CNF/EP presented a broad interphase with a gradual, gradient-like modulus reduction. Acting as a “stress-transfer bridge”, this gradient interphase with continuous modulus distribution mitigates the abrupt property mismatch between fibers and the matrix, facilitates smooth stress transmission over the interface, suppresses local stress concentration, and therefore greatly strengthens interfacial bonding. It should be noted that the transition layer thickness measured by EDS reflects the zone of elemental interdiffusion between the fibers and the matrix; this zone is typically larger than the “effective modulus transition thickness” derived from AFM-PF-QNM, as the latter is more sensitive to local changes in the mechanical properties of the rigid CNT network. Both metrics consistently confirm the formation of a thick, gradient-bearing interface layer.

**Figure 13:**
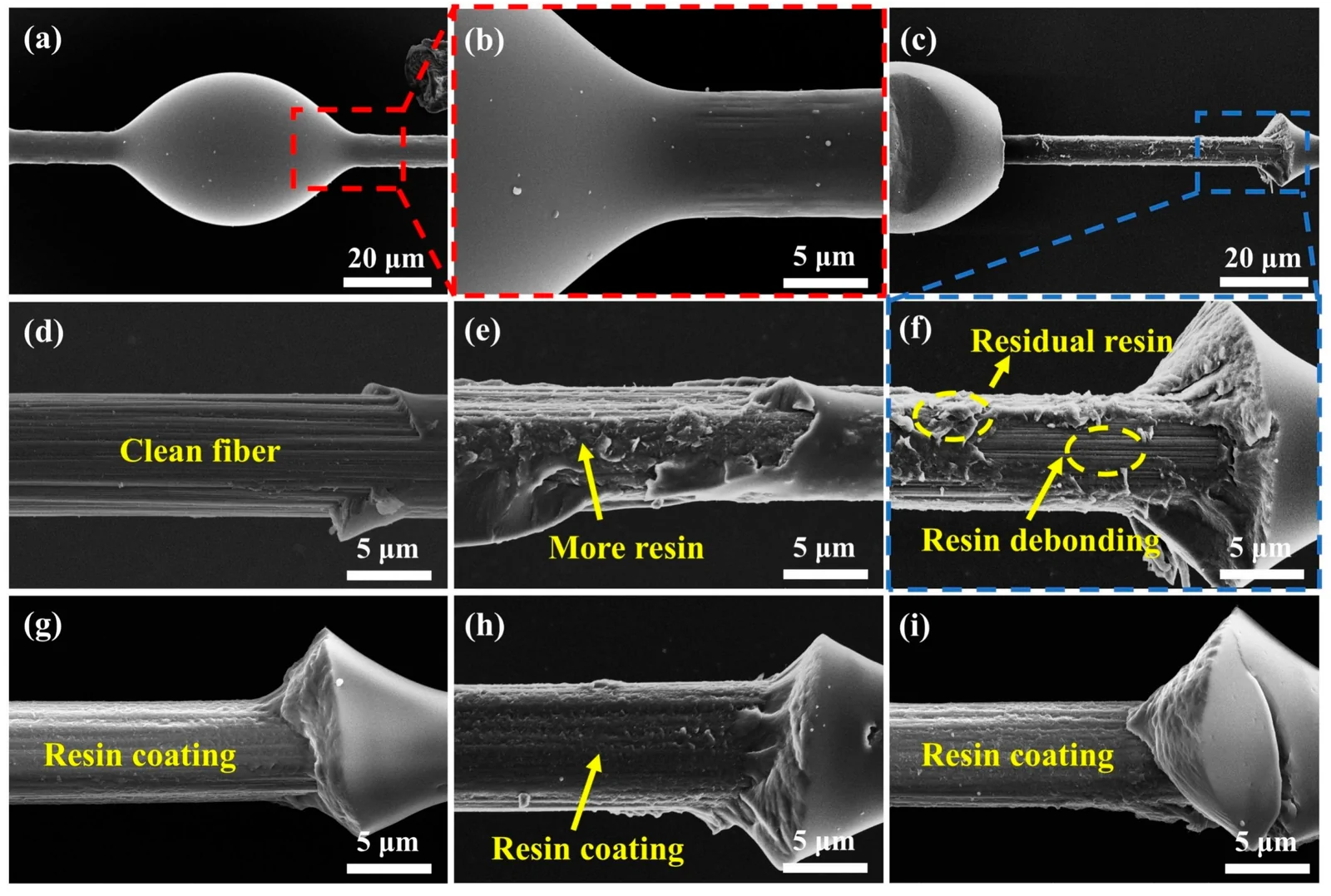
Surface morphologies of epoxy resin droplets on carbon fibers (**a**,**b**) and fracture morphologies of detached droplets: (**d**) CF, (**e**) CF-0.1%CNT, (**c**,**f**) CF-PDA-CNF, (**g**) CF-0.05%CNT-PDA-CNF, (**h**) CF-0.1%CNT-PDA-CNF, and (**i**) CF-0.2%CNT-PDA-CNF.

The debonded surface morphologies after fiber pull-out tests were characterized to assess the interfacial bonding strength of different modified fibers. As illustrated in Figure 13a,b, the resin micro-droplet remains intact and fully coats the carbon fiber. In Figure 13d, following delamination, the CF surface displays an absence of residual resin, indicative of a typical instance of interfacial delamination failure. Figure 13e shows that, after debonding, CF-0.1%CNT exhibits a significant presence of residual resin on its surface with an uneven distribution, indicating its superior interfacial bonding compared to CF. In Figure 13c,f, a minimal quantity of resin residue can be observed following the debonding of the CF-PDA-CNF/EP composite, with some fiber surfaces exposed. For the modified composite material (CF-xCNT-PDA-CNF, Figure 13g–i), after fiber debonding, uniform resin coating on the fiber surface can be observed. The resin morphology exhibits coarse wrinkles due to shear deformation, suggesting enhanced interfacial strength.

**Figure 14:**
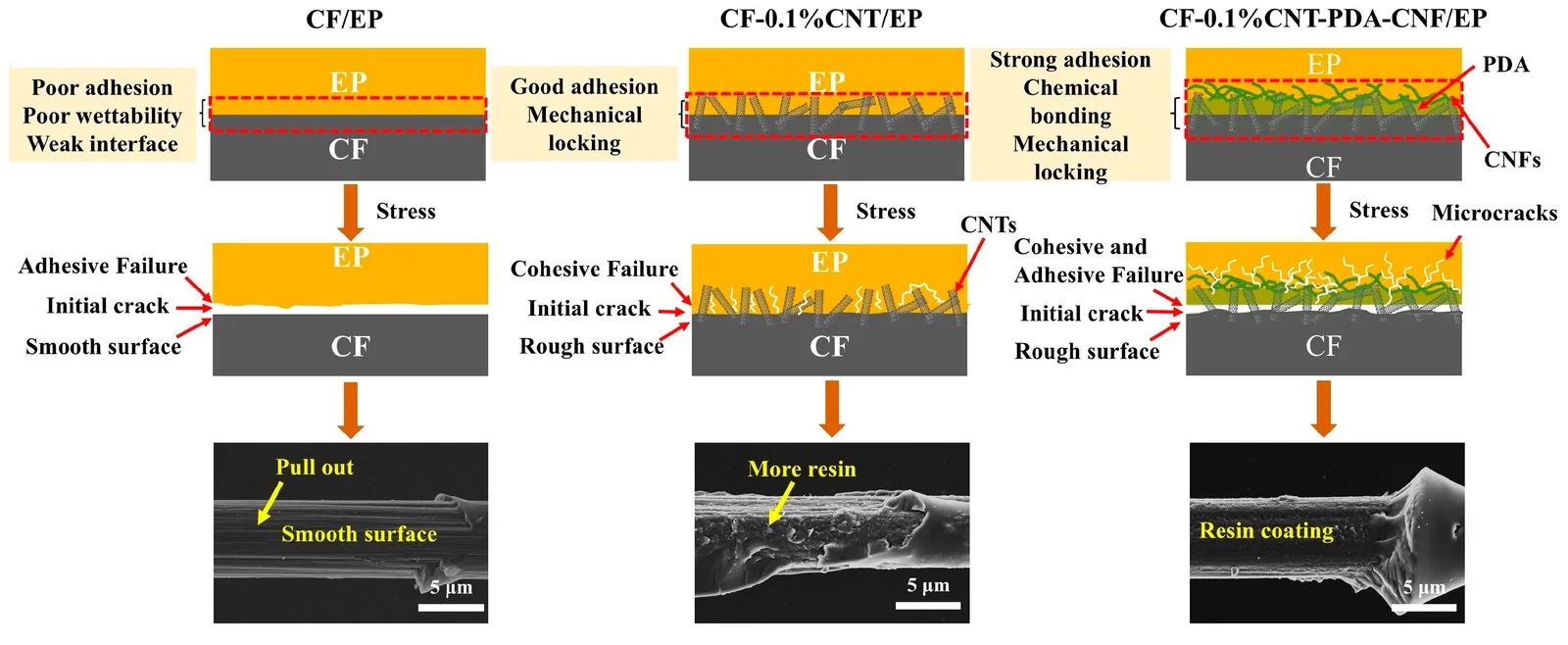
Schematic diagram of reinforcement mechanisms at the interfaces of carbon-fiber-reinforced composites.

Figure 14 illustrates the interfacial reinforcement mechanism of the “three-dimensional nanospider web” structure. CNTs constitute the rigid component, forming a high-modulus “rigid protrusion network” on carbon-fiber surfaces that acts as a load-transfer bridge for stress transmission and a physical barrier suppressing crack initiation and propagation. For the flexible components, PDA functions as a critical anchoring intermediate layer; it immobilizes CNTs and CNFs onto the chemically inert carbon-fiber surface through covalent and non-covalent interactions while introducing abundant polar functional groups, yet PDA alone is incapable of building the extended nanonetwork needed for a gradient interphase. As flexible one-dimensional building blocks, CNFs bridge adjacent CNTs to generate continuous flexible network segments and endow the interphase with compliant characteristics; nevertheless, CNFs cannot achieve stable attachment onto pristine carbon fibers without PDA-mediated adhesion. Accordingly, neither PDA nor CNFs alone can realize the target interphase architecture.

The “rigid–flexible” CNT-PDA-CNF architecture on the carbon-fiber surface firmly anchors both CNTs and CNFs to the resin matrix via covalent bonds formed by PDA’s active groups. This chemical linkage network enhances the interfacial energy dissipation capacity. The rigid component provides structural support at the interface, while the flexible component enhances toughness. The two components transition through a modulus gradient, optimizing the interface bonding between the carbon fibers and the resin. When composite materials are subjected to external forces, CNTs first inhibit the initiation of macroscopic cracks, while the flexible PDA-CNF dissipates localized impact energy through deformation. The synergistic action between the CNT “bridging effect” and the PDA-CNF “crack deflection” function effectively impedes crack propagation upon initiation. This shifts the fracture mode from pure adhesive failure to a mixed adhesive–cohesive one. By first arresting and then dissipating crack energy, this mechanism enables the composite to achieve a simultaneous enhancement in both interfacial strength and toughness.

## 4. Conclusions

In this study, a “three-dimensional nanospider web” structure was constructed on carbon-fiber surfaces by CNTs, PDA, and CNFs. Different from conventional rigid–flexible interphase designs, this hierarchical nanostructure successfully achieves interfacial thickness enlargement and forms a continuous and smooth modulus gradient interphase between carbon fibers and an epoxy matrix. This architecture substantially increases fiber surface roughness, and promotes chemical bonding via active functional groups (-NH_2_, -COOH, -OH). Consequently, interfacial properties are significantly enhanced, which contributes to notable enhancements in the composites’ overall mechanical performances. The IFSS (95.71 MPa), ILSS (73.19 MPa), tensile strength (701.08 MPa), and flexural strength (934.41 MPa) of the CF-0.1%CNT-PDA-CNF/EP composite material increased by 38.39%, 54.93%, 51.74%, and 64.98%, respectively, compared to the composite made from desized carbon fibers. Benefiting from the optimized interfacial structure, the continuous modulus gradient effectively eliminates the abrupt modulus mismatch at the fiber–matrix interface, guides stress transfer from the matrix to fibers uniformly, suppresses local stress concentration, and extends crack propagation paths, thereby substantially improving the interfacial adhesion properties of the composite material. This study achieved synergistic regulation of chemical bonding, mechanical interlocking, and modulus transition at the fiber–matrix interface through the development of a reticular “rigid–flexible” modulus transition layer on the carbon-fiber surface. The innovative dual optimization of interfacial thickness and modulus gradient endows the composite with superior mechanical performance, providing a novel and feasible strategy for the structural design and mechanical property optimization of high-performance carbon-fiber composites.

## Figures and Tables

**Table 1:** Surface element content of carbon-fiber samples obtained by different modification methods.

Sample Name	Chemical Element Content (at.%)		
	C	O	N
CF	69.58	30.42	0
CF-0.1%CNT	73.04	26.96	0
CF-0.1%CNT-PDA-CNF	60.81	35.08	4.11

## Data Availability

The original contributions presented in the study are included in the article. Further inquiries can be directed to the corresponding authors.
